# Comparative evaluation of RANKL and OPG levels in gingival crevicular fluid and saliva during orthodontic treatment using thermoformed and direct-printed aligners

**DOI:** 10.1590/2177-6709.31.3.e2625300.oar

**Published:** 2026-08-07

**Authors:** Pooja LAKSHMANAN, Shivakumar BASKARAN, Swarna ALAMELU, Karthick BASKAR

**Affiliations:** 1 Ragas Dental College and Hospital, Department of Periodontics (Tamil Nadu, India).

**Keywords:** Orthodontic tooth movement, RANKL, OPG, Clear plastic aligners, Gingival crevicular fluid, Movimentação dentária ortodôntica, RANKL, OPG, Alinhadores transparentes, Fluido crevicular gengival

## Abstract

**Introduction:**

Orthodontic tooth movement (OTM) is regulated by alveolar bone remodeling through the receptor activator of nuclear factor kappa-B ligand (RANKL) and osteoprotegerin (OPG) axis. Clear plastic aligners (CPAs), available as thermoformed and direct-printed variants, differ in force delivery and material properties, influencing biomarker responses.

**Objective:**

This prospective pilot randomized study compared salivary and gingival crevicular fluid (GCF) levels of RANKL and OPG during treatment with thermoformed and direct-printed aligners, and evaluated correlations between saliva and GCF.

**Material and Methods:**

Eight healthy adults (18-40 years) requiring orthodontic treatment were randomly assigned to thermoformed or direct-printed aligners. GCF and unstimulated saliva were collected at baseline and at 1, 2, 3, 4, and 6 weeks. RANKL and OPG were quantified using ELISA, and data were analyzed with SPSS v21.0 (IBM, USA).

**Results:**

Thermoformed aligners exhibited a peak in GCF RANKL at 3 weeks, followed by a decline at 6 weeks. Direct-printed aligners showed a gradual reduction, with significantly lower RANKL at 4 and 6 weeks (p < 0.05). OPG levels were consistently higher in the direct-printed group across all time points in saliva and GCF (p < 0.05). Salivary OPG strongly correlated with GCF OPG at baseline, 3, and 6 weeks, while RANKL correlations were higher during later treatment phases.

**Conclusion:**

Aligner types elicited biomarker responses consistent with bone remodeling. Direct-printed aligners demonstrated a trend toward a more stable, OPG-dominant biological response. These findings should be interpreted cautiously, due to the pilot nature of the study. Saliva correlated with GCF, supporting its use as a non-invasive biomarker source. Larger longitudinal studies are warranted.

## RESUMO

**Introdução:**

A movimentação dentária ortodôntica (MDO) é regulada pela remodelação óssea alveolar através do eixo do ligante do receptor-ativador do fator nuclear kappa-B (RANKL) e da osteoprotegerina (OPG). Os alinhadores transparentes (ATs), disponíveis em versões termoformadas e impressas diretamente, diferem na aplicação da força e nas propriedades do material, influenciando as respostas dos biomarcadores.

**Objetivo:**

Este estudo piloto prospectivo randomizado comparou os níveis de RANKL e OPG na saliva e no fluido crevicular gengival (FCG) durante o tratamento com alinhadores termoformados e impressos diretamente, e avaliou as correlações entre a saliva e o FCG.

**Material e Métodos:**

Oito adultos saudáveis (18-40 anos) que necessitavam de tratamento ortodôntico foram aleatoriamente designados para usar alinhadores termoformados ou impressos diretamente. O FCG e a saliva não estimulada foram coletados no início do estudo e após 1, 2, 3, 4 e 6 semanas. Os níveis de RANKL e OPG foram quantificados por ELISA e os dados foram analisados com o SPSS versão 21.0 (IBM, EUA).

**Resultados:**

Os alinhadores termoformados apresentaram um pico de RANKL no FCG em 3 semanas, seguido por um declínio em 6 semanas. Os alinhadores impressos diretamente mostraram uma redução gradual, com níveis de RANKL significativamente menores em 4 e 6 semanas (p < 0,05). Os níveis de OPG foram consistentemente mais altos no grupo de impressão direta em todos os momentos de avaliação, tanto na saliva quanto no FCG (p < 0,05). A OPG salivar apresentou forte correlação com a OPG no FCG no início do estudo, em 3 e 6 semanas, enquanto as correlações com RANKL foram maiores durante as fases posteriores do tratamento.

**Conclusão:**

Ambos os tipos de alinhadores induziram respostas de biomarcadores consistentes com a remodelação óssea. Os alinhadores impressos diretamente demonstraram uma tendência a uma resposta biológica mais estável e com predominância de OPG. Esses achados devem ser interpretados com cautela, devido à natureza piloto do estudo. A saliva apresentou correlação com o FCG, corroborando seu uso como fonte não invasiva de biomarcadores. Estudos longitudinais com amostras maiores são necessários.

## INTRODUCTION

The periodontium is a dynamic unit that supports teeth, distributes occlusal forces, and preserves dentoalveolar stability through continuous remodeling by osteoclasts and osteoblasts. Alveolar bone adjusts to forces during orthodontic tooth movement (OTM), where resorption occurs on the side under pressure and apposition, on the side under tension.^1^ Bone remodelling is regulated by local mediators including growth factors and cytokines, as well as systemic hormones.^2^ Orthodontic success depends on periodontal health, optimal force application, and plaque control. Mechanical forces trigger cellular and molecular cascades measurable through biomarkers in gingival crevicular fluid (GCF) and saliva.^3^

Among these, RANKL (receptor activator of nuclear factor kappa-B ligand) and OPG (osteoprotegerin), play a central role in bone remodeling. RANKL stimulates osteoclastogenesis, while OPG inhibits it by acting as a decoy receptor.^4^ The RANKL/OPG ratio is important for bone turnover and serves as a biomarker for both OTM and periodontal disease.

Clear plastic aligners (CPAs) have transformed Orthodontics by applying controlled forces through removable trays, offering improved esthetics, comfort, and hygiene over fixed appliances. Thermoformed aligners are fabricated by heating plastic sheets and molding them over 3D-printed dental models. Their thickness and elasticity often vary due to the forming process, which may influence force consistency. Direct-printed aligners, produced by 3D-printing biocompatible resins directly into the aligner form, bypass the intermediate model stage, improving dimensional accuracy, reproducibility, and customization. Direct printing also allows precise control of thickness distribution, which may yield more predictable force application.^5^ ^,^ ^6^

Although clear aligners are widely used in Orthodontics, limited evidence exists regarding how different aligner fabrication methods influence biological responses during orthodontic tooth movement. Thermoformed and direct-printed aligners differ in material properties and force delivery, which may affect bone remodeling and inflammatory mediator expression. However, comparative biological evidence supporting these differences is scarce. Therefore, this study aimed to compare the levels of RANKL and OPG in GCF and saliva during orthodontic treatment using thermoformed and direct-printed aligners, and to evaluate the correlation between salivary and GCF biomarkers.

## MATERIAL AND METHODS

### STUDY DESIGN AND ETHICAL APPROVAL

This investigation was conducted as a prospective, randomized pilot clinical study to evaluate biological responses associated with different clear aligner fabrication techniques during orthodontic tooth movement. Randomization was performed to explore preliminary biological trends and feasibility, rather than to establish definitive clinical superiority between aligner systems. The study protocol was approved by the Institutional Ethics Committee (Ref No: EC/NEW/INST/2023/4006) and conducted in accordance with the Declaration of Helsinki. The study was registered with the Clinical Trials Registry of India (CTRI/2024/09/074517).

All participants were informed about the study objectives and procedures, and written informed consent was obtained prior to enrollment. Participation was voluntary and did not influence orthodontic treatment outcomes.

### STUDY POPULATION

Eight systemically healthy adult patients aged 18-40 years who required orthodontic treatment were recruited from the Department of Orthodontics and referred to the Department of Periodontics for periodontal evaluation. All participants underwent comprehensive periodontal examination before inclusion, to confirm gingival and periodontal health.

Supragingival oral prophylaxis was performed prior to baseline sample collection, and plaque and bleeding scores were recorded to ensure optimal oral hygiene status throughout the study period. Baseline periodontal and clinical parameters were comparable across participants, and no clinically relevant differences were observed between groups at enrollment.

### INCLUSION CRITERIA

Systemically healthy individuals with good oral hygiene (Plaque Index < 20%).

Angle’s Class I malocclusion with mild to moderate spacing, crowding, or rotation not requiring tooth extraction.

Objective malocclusion severity classified as Index of Orthodontic Treatment Need (IOTN) grade 2-3.

No prior orthodontic treatment or craniofacial abnormalities.

### EXCLUSION CRITERIA

Age < 18 years or intake of medications affecting bone metabolism in the preceding six months.

Systemic conditions (e.g., diabetes, osteoporosis), pregnancy, lactation, smoking, or tobacco use.

Use of anti-inflammatory drugs or medicated mouthwashes during the study period.

### SAMPLE SIZE JUSTIFICATION

As this investigation was conducted as a pilot study to generate preliminary data and assess biological trends, a formal sample size calculation was not performed. Consistent with previous pilot studies evaluating biomarker changes during orthodontic tooth movement, a total sample size of eight participants was considered sufficient to estimate effect sizes, assess feasibility, and guide the design of future large-scale randomized clinical trials.⁷

### GROUPING AND ALIGNER FABRICATION

Participants were randomly allocated into two groups using a simple randomization method, with four subjects in each group:

Group 1: Thermoformed clear plastic aligners (Invisalign^®^, Align Technology, USA).

**Figure 1:**
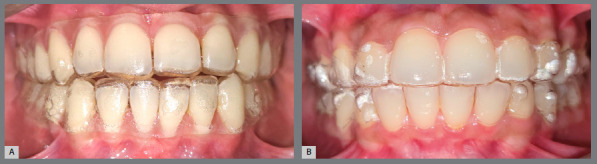
A) Group 1: Thermoformed clear plastic aligner (CPA). B) Group 2: Direct printed CPA.

Group 2: Direct-printed clear plastic aligners (Graphy^®^, TA28 Resin, Graphy Inc., Seoul, South Korea) (Fig. 1)

To minimize biological and mechanical variability, only patients with mild to moderate malocclusion not requiring extractions were included. Aligner treatment plans were digitally designed using standardized staging protocols, and no auxiliary attachments or additional mechanics were used during the observation period.

All participants were instructed to wear their aligners according to standard orthodontic protocols, with identical daily wear duration and aligner change intervals across both groups. This standardization was intended to ensure comparable orthodontic force application and isolate the biological effects related to aligner fabrication technique.

### SAMPLE COLLECTION

Baseline samples of GCF and unstimulated whole saliva (UWS) were collected before treatment. Subsequent samples were collected at aligner change intervals: Day 0 (baseline), Day 7 (week 1), Day 14 (week 2), Day 21 (week 3), Day 28 (week 4), and Day 42 (week 6).^7^

### GCF COLLECTION

Patients underwent scaling 24 hours before sampling. GCF samples were obtained from the mesiobuccal sulcus of the maxillary right first premolar in all participants, a site chosen for accessibility and minimal saliva contamination. The tooth was isolated using cotton rolls, and GCF was collected using a 0-5 μL microcapillary pipette from the sulcus entrance for up to 10 minutes. Approximately 2 μL of GCF was obtained per site, eluted in 200 μL phosphate-buffered saline, and preserved at −80°C until analysis.^8^

### SALIVA COLLECTION

Unstimulated saliva was collected using the Modified Navazesh protocol.^9^ Patients avoided food/drink for one hour before sampling, rinsed with water, and were seated with the head slightly forward, to allow passive drooling into sterile containers for 5 minutes. Samples were preserved at −80°C until analysis. All collections were performed at consistent times of day, to reduce circadian variation.

### ELISA ASSAY

RANKL and OPG levels were measured using the Human RANKL ELISA Kit (KTE62865, Abbkine, China) and Human OPG ELISA Kit (KTE60224, Abbkine, USA), following the manufacturer’s instructions.^10^ Each sample was assayed in duplicate, to ensure accuracy and reproducibility. Results were expressed in pmol/L for RANKL and pg/mL for OPG. 

### STATISTICAL ANALYSIS

Statistical analysis was performed using SPSS version 21.0 (IBM, USA). Data normality was assessed using the Kolmogorov-Smirnov test. Intergroup comparisons were performed using ANOVA or Kruskal-Wallis tests, as appropriate. Correlations were assessed using Pearson or Spearman correlation coefficients, based on data distribution. Given the pilot nature of the study and the limited sample size, analyses were exploratory and focused on identifying temporal trends rather than drawing definitive inferential conclusions. P values ≤ 0.05 were considered statistically significant.

## RESULTS

### RANKL EXPRESSION IN GCF AND SALIVA

RANKL expression levels in GCF and saliva for both thermoformed and direct-printed aligner groups are presented in Table 1.

**Table 1:** Expression and comparison of RANKL (Receptor activator of nuclear factor kappa-B ligand ) levels in gingival crevicular fluid (GCF) and saliva between thermoformed and direct-printed aligner groups.

Time point	GCF Thermoformed (mean ± SD, pmol/L)	GCF Direct-printed (mean ± SD, pmol/L)	Sig (p)	Saliva Thermoformed (mean ± SD, pmol/L)	Saliva Direct-printed (mean ± SD, pmol/L)	Sig (p)
Baseline	267.94 ± 68.19	278.82 ± 13.65	0.801	242.29 ± 22.98	185.77 ± 13.71	0.013*
1 week	268.21 ± 12.33	254.57 ± 24.82	0.376	235.77 ± 55.72	158.07 ± 12.12	0.068
2 week	251.62 ± 35.52	242.37 ± 22.42	0.712	206.40 ± 25.91	206.00 ± 13.88	0.980
3 week	316.21 ± 53.90	283.79 ± 17.03	0.370	238.61 ± 74.92	201.99 ± 20.63	0.457
4 week	254.37 ± 50.61	181.03 ± 25.57	0.043*	206.37 ± 35.84	196.61 ± 24.15	0.703
6 week	236.53 ± 14.61	210.98 ± 23.82	0.036*	223.28 ± 20.49	221.82 ± 45.65	0.480

*Significant at p ≤ 0.05.

In the GCF, the thermoformed group showed relatively stable RANKL levels from baseline to week 2, followed by a notable peak at week 3 (316.21 ± 53.90 pmol/L) and a decline at week 6 (236.53 ± 14.61 pmol/L). In contrast, the direct-printed group demonstrated a gradual reduction from baseline (278.82 ± 13.65 pmol/L) to week 4 (181.03 ± 25.57 pmol/L), with partial recovery at week 6 (210.98 ± 23.82 pmol/L).

Significant intergroup differences were observed at week 4 (p = 0.043) and week 6 (p = 0.036), indicating lower GCF RANKL levels in the direct-printed group during the later phase of observation.

In saliva, RANKL levels in the thermoformed group fluctuated slightly, with a dip at week 2 (206.40 ± 25.91 pmol/L) and recovery by week 6 (223.28 ± 20.49 pmol/L). The direct-printed group, however, exhibited lower baseline RANKL values (185.77 ± 13.71 pmol/L) and a gradual rise throughout the study period (221.82 ± 45.65 pmol/L at week 6). A statistically significant difference was observed at baseline (p = 0.013).

### OPG EXPRESSION IN GCF AND SALIVA

The expression levels of OPG in GCF and saliva are shown in Table 2.

**Table 2:** Expression and comparison of osteoprotegerin ( OPG ) levels in gingival crevicular fluid (GCF) and saliva between thermoformed and direct-printed aligner groups.

Time point	GCF Thermoformed (mean ± SD, pg/mL)	GCF Direct-printed (mean ± SD, pg/mL)	Sig (p)	Saliva Thermoformed (mean ± SD, pg/mL)	Saliva Direct-printed (mean ± SD, pg/mL)	Sig (p)
Baseline	406.63 ± 59.13	429.36 ± 50.05	0.542	372.27 ± 41.24	439.48 ± 95.79	0.033*
1 week	339.79 ± 24.55	482.43 ± 46.76	0.002*	327.56 ± 53.80	489.49 ± 94.10	0.024*
2 week	423.25 ± 53.09	534.55 ± 26.21	0.022*	329.46 ± 59.62	451.50 ± 102.30	0.001*
3 week	484.73 ± 44.44	523.10 ± 70.46	0.045*	343.64 ± 59.68	456.28 ± 55.44	0.019*
4 week	395.85 ± 19.43	469.45 ± 35.13	0.024*	366.06 ± 46.69	504.77 ± 74.26	0.028*
6 week	338.83 ± 23.58	453.60 ± 71.89	0.002*	375.49 ± 24.41	520.23 ± 99.15	0.034*

*Significant at p ≤ 0.05.

In the GCF, the thermoformed group exhibited a decline in OPG concentration at week 1 (339.79 ± 24.55 pg/mL), followed by a peak at week 3 (484.73 ± 44.44 pg/mL). The direct-printed group demonstrated consistently higher OPG levels across all time points, reaching a maximum at week 2 (534.55 ± 26.21 pg/mL). Significant intergroup differences were observed at all time points, except baseline (p ≤ 0.045).

In saliva, the thermoformed group displayed relatively stable OPG levels (range = 327.56-375.49 pg/mL), whereas the direct-printed group showed markedly higher concentrations throughout, with a peak at week 6 (520.23 ± 99.15 pg/mL). Intergroup differences were statistically significant at all time points (p ≤ 0.034).

### CORRELATION BETWEEN GCF AND SALIVARY LEVELS

Correlation analysis between GCF and salivary levels of RANKL and OPG is summarized in Table 3.

**Table 3:** Correlation between GCF and salivary levels (Pearson r, p value).

Marker	Group	Baseline	3 week	6 week
RANKL	Thermoformed	r = 0.57, P = 0.07	r = 0.61, P = 0.04	r = 0.68, P = 0.00
	Direct printed	r = 0.23, P = 0.08	r = 0.67, P = 0.06	r = 0.72, P = 0.03
OPG	Thermoformed	r = 0.42, P = 0.01	r = 0.55, P = 0.00	r = 0.68, P = 0.00
	Direct printed	r = 0.48, P = 0.01	r = 0.62, P = 0.00	r = 0.70, P = 0.00

RANKL = Receptor activator of nuclear factor kappa-B ligand. OPG = Osteoprotegerin. GCF = Gingival crevicular fluid.

For RANKL, significant positive correlations were noted in the thermoformed group at week 3 (r = 0.61, p = 0.04) and week 6 (r = 0.68, p < 0.001). In the direct-printed group, correlation became significant at week 6 (r = 0.72, p = 0.03).

For OPG, both groups demonstrated strong positive correlations at baseline, week 3, and week 6 (r = 0.42-0.70, p ≤ 0.01), indicating that salivary OPG reliably reflects corresponding GCF levels.

## DISCUSSION

The alveolar bone undergoes continuous remodeling through the coordinated activity of osteoblasts and osteoclasts, thereby maintaining skeletal integrity in accordance with Wolff’s law. Orthodontic force application disrupts periodontal homeostasis by generating areas of tension and compression within the periodontal ligament (PDL) and surrounding alveolar bone. These mechanical stresses induce vascular alterations and a sterile inflammatory response, resulting in the release of molecular mediators that regulate bone remodeling. Among these, the RANK/RANKL/OPG signaling axis plays a pivotal role: RANKL, produced by osteoblasts and PDL cells, promotes osteoclast differentiation and bone resorption; whereas OPG, secreted by fibroblasts, endothelial cells, and PDL cells, acts as a decoy receptor that inhibits osteoclastogenesis. Consequently, the RANKL/OPG ratio is critical in maintaining the balance of bone metabolism during orthodontic tooth movement.^11^

This pilot study evaluated the expression of RANKL and OPG in saliva and GCF during orthodontic treatment using thermoformed and direct-printed aligners across six time points, over a six-week period. In the thermoformed aligner group, GCF RANKL levels peaked at week 3, consistent with its established role in osteoclast activation during orthodontic force application.^12^ Transient upregulation of RANKL during the early to mid-treatment phase reflects active bone remodeling and aligns with previous experimental and clinical studies demonstrating enhanced osteoclastic activity during orthodontic tooth movement.^13^ ^,^ ^14^

In contrast, direct-printed aligners demonstrated a gradual reduction in GCF RANKL levels after baseline, with significantly lower concentrations observed at weeks 4 and 6. This finding may be attributed to differences in force delivery characteristics, as direct-printed aligners are reported to provide more controlled and uniform force application, compared to thermoformed appliances.^6^ ^,^ ^15^ Previous investigations have demonstrated favorable force profiles, movement predictability, and biomechanical consistency in direct-printed aligners, which may contribute to reduced periodontal stress and modulation of inflammatory mediator release.^6^ ^,^ ^15^

In saliva, thermoformed aligners showed higher baseline RANKL levels, which may be attributed to individual biological variability or aligner-specific responses, particularly during the early treatment phase.

OPG expression demonstrated marked intergroup differences. In GCF, thermoformed aligners exhibited a peak in OPG levels at three weeks, likely representing a compensatory response to increased RANKL expression.^13^ In contrast, direct-printed aligners maintained consistently higher OPG levels across all time points, suggesting a trend toward a more protective, bone-preserving biological response.^12^ This observation is consistent with the established inhibitory role of OPG in osteoclastogenesis and bone resorption.^11^ ^,^ ^12^ Salivary OPG levels reinforced these trends, with the direct-printed aligner group exhibiting significantly higher concentrations throughout the study period. Elevated salivary OPG has been proposed as a potential indicator of periodontal stability and balanced bone remodeling activity.^3^

Overall, the observed RANKL/OPG dynamics suggest that direct-printed aligners may promote a more balanced bone remodeling environment, potentially reducing the risk of adverse outcomes such as excessive bone resorption or root resorption, while still permitting effective orthodontic tooth movement. Similar associations between increased OPG expression and regulation of bone remodeling during orthodontic tooth movement have been reported previously.^2^ ^,^ ^12^ However, these interpretations should be considered preliminary, due to the pilot nature of the study and the limited sample size.

Correlation analysis between GCF and salivary biomarkers further highlighted the diagnostic reliability of oral fluids. For RANKL, significant correlations were observed in the thermoformed aligner group at weeks 3 and 6, and in the direct-printed aligner group, at week 6, indicating that salivary RANKL may reflect localized periodontal changes during active and later phases of orthodontic treatment.^3^ For OPG, both groups demonstrated significant correlations at baseline, week 3, and week 6, suggesting that salivary OPG more consistently mirrors GCF levels than RANKL, and may serve as a reliable indicator of bone remodeling activity.^3^ The stability of OPG correlations across fluids supports earlier reports of its protective role against excessive bone resorption in both orthodontic and periodontal contexts.^11^ ^,^ ^12^

Collectively, these findings emphasize saliva as a practical, non-invasive surrogate for GCF, particularly for monitoring OPG-related protective mechanisms during orthodontic treatment. Previous studies evaluating oral-fluid biomarkers have similarly supported the utility of saliva as a diagnostic medium for monitoring biological responses associated with orthodontic tooth movement and periodontal remodeling.^3^

## LIMITATIONS

The current study included a limited sample size (n = 8), which restricts the statistical power and generalizability of the findings. Accordingly, the results should be interpreted with caution. This investigation was designed as a pilot study to assess preliminary biological differences between direct-printed and thermoformed aligners. Future studies with larger sample sizes, longer follow-up durations, and comprehensive clinical outcome measures are recommended to validate these preliminary observations and strengthen the conclusions.

## CONCLUSION

This study reinforces the importance of the RANKL/OPG axis in orthodontically induced bone remodeling. Both thermoformed and direct-printed aligners elicited molecular responses consistent with tooth movement. Within the limitations of this pilot study, direct-printed aligners demonstrated a more stable, OPG-dominant biomarker profile, suggesting a potential trend toward more controlled biological responses rather than definitive clinical superiority. Saliva proved to be a reliable surrogate for gingival crevicular fluid, supporting its potential role in non-invasive biomarker monitoring. These preliminary findings warrant confirmation through larger, long-term randomized clinical studies.

## Data Availability

All data generated or analyzed during this study are included in this published article.

## References

[1] Li Y, Zhan Q, Bao M, Yi J, Li Y (2021). Biomechanical and biological responses of periodontium in orthodontic tooth movement update in a new decade. Int J Oral Sci.

[2] Grant M, Wilson J, Rock P, Chapple I (2013). Induction of cytokines, MMP9, TIMPs, RANKL and OPG during orthodontic tooth movement. Eur J Orthod.

[3] Gaur A, Maheshwari S, Verma SK (2017). Detection of molecular biomarkers as a diagnostic tool in the planning and progression of orthodontic treatment. J Orofac Sci.

[4] Aldahlawi S (2019). Evaluation of serum RANKL and OPG concentrations in patients with periodontitis in Saudi Arabia. Int J Pharm Res Allied Sci.

[5] Ludwig B, Ojima K, Schmid JQ, Knode V, Nanda R (2024). Direct-printed aligners a clinical status report. J Clin Orthod.

[6] Hertan E, McCray J, Bankhead B, Kim KB (2022). Force profile assessment of direct-printed aligners versus thermoformed aligners and the effects of non-engaged surface patterns. Prog Orthod.

[7] Baeshen HA (2022). GCF cytokine profile comparison between patients with lingual fixed appliances and aligners. J King Saud Univ Sci.

[8] Bergamo AZN, Nelson-Filho P, do Nascimento C, Casarin RCV, Casati MZ (2018). Cytokine profile changes in gingival crevicular fluid after placement of different bracket types. Arch Oral Biol.

[9] Navazesh M, Christensen CM (1982). A comparison of whole mouth resting and stimulated salivary measurement procedures. J Dent Res.

[10] Hatiboglu S, Yanar F, Ozturk A, Basar Y, Aydogan M, Ozkok E (2024). Peroxisome proliferator-activated receptor gamma and osteoprotegerin levels as an indicator and diagnostic predictor of endothelial dysfunction. Turk J Biochem.

[11] Boyce BF, Xing L (2008). Functions of RANKL/RANK/OPG in bone modeling and remodeling. Arch Biochem Biophys.

[12] Yamaguchi M (2009). RANK/RANKL/OPG during orthodontic tooth movement. Orthod Craniofac Res.

[13] Kanzaki H, Chiba M, Takahashi I, Haruyama N, Nishimura M, Mitani H (2004). Local OPG gene transfer to periodontal tissue inhibits orthodontic tooth movement. J Dent Res.

[14] Jiang Y, Guan Y, Lan Y, Chen S, Li T, Zou S (2021). Mechanosensitive Piezo1 in periodontal ligament cells promotes alveolar bone remodeling during orthodontic tooth movement. Front Physiol.

[15] Migliorati M, Drago S, Castroflorio T, Pesce P, Battista G, Campobasso A (2024). Accuracy of orthodontic movements with 3D printed aligners A prospective observational pilot study. Korean J Orthod.

